# Supplementary figures and images for: Diagnostic ability of confocal scanning ophthalmoscope for the detection of concurrent retinal disease in eyes with asteroid hyalosis
Source: PLoS One. 2024 Dec 5;19(12):e0306091. doi: 10.1371/journal.pone.0306091 (PMC11620638; doi:10.1371/journal.pone.0306091)

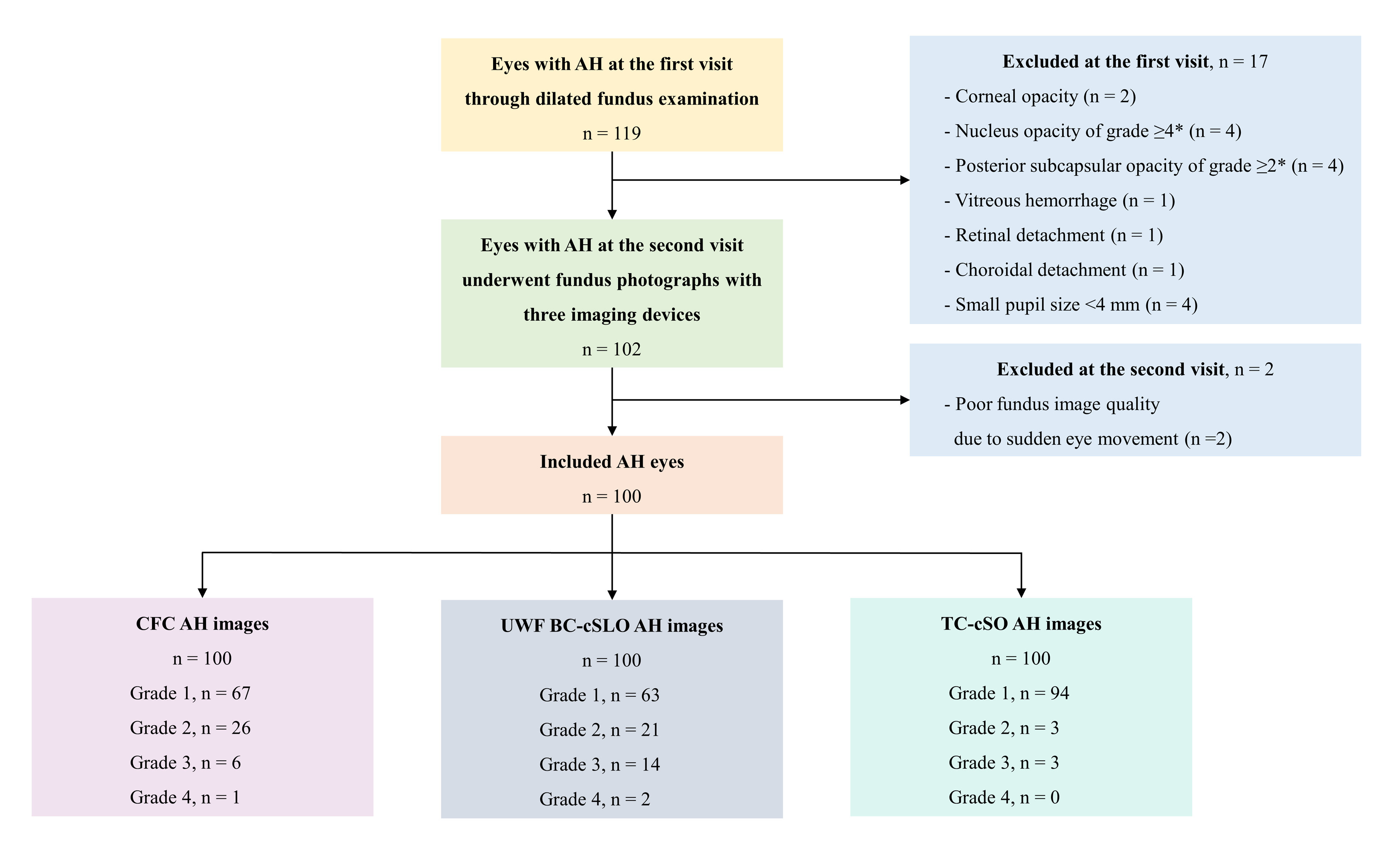

Supplement: S1 Fig — AH, Asteroid hyalosis; BC-cSLO, Bicolor confocal scanning laser ophthalmoscope; TC-cSO, True-color confocal scanning ophthalmoscope; UWF, ultra-widefield. * = According to the Lens Opacities Classification System III classification. (TIF) [file pone.0306091.s001.tif]
