# Supplementary material for: Diagnostic ability of confocal scanning ophthalmoscope for the detection of concurrent retinal disease in eyes with asteroid hyalosis
Source: PLoS One. 2024 Dec 5;19(12):e0306091. doi: 10.1371/journal.pone.0306091 (PMC11620638; doi:10.1371/journal.pone.0306091)
Supplement: S1 Table — BC-cSLO, Bicolor confocal scanning laser ophthalmoscope; CFC, Color fundus camera; TC-cSO, True-color confocal scanning ophthalmoscope. * Grade of obscuration was defined as minimal for Grade 1 and significant for Grades 2–4. (DOCX) [file pone.0306091.s002.docx]

**S2 Table. Differences in the Diagnostic Ability of Each Imaging Device According to the Grade of Obscuration in Color Fundus Camera.**

| **Imaging Device** | **Grade of Obscuration**^*^ **in CFC** | |
| --- | --- | --- |
| **CFC** | **Minimal Obscuration** | **Significant Obscuration** |
| Accuracy (%) | 90.0 | 86.9 |
| Sensitivity (%) | 51.4 | 24.2 |
| Specificity (%) | 98.1 | 98.9 |
| Precision (%) | 85.2 | 81.6 |
| F1 score | 0.641 | 0.373 |
| **BC-cSLO** | **Minimal Obscuration** | **Significant Obscuration** |
| Accuracy (%) | 89.3 | 86.7 |
| Sensitivity (%) | 45.0 | 25.8 |
| Specificity (%) | 98.6 | 98.5 |
| Precision (%) | 87.5 | 76.7 |
| F1 score | 0.594 | 0.386 |
| **TC-cSO** | **Minimal Obscuration** | **Significant Obscuration** |
| Accuracy (%) | 95.1 | 93.6 |
| Sensitivity (%) | 83.6 | 75.0 |
| Specificity (%) | 97.5 | 97.1 |
| Precision (%) | 87.6 | 83.5 |
| F1 score | 0.856 | 0.790 |

BC-cSLO, Bicolor confocal scanning laser ophthalmoscope; CFC, Color fundus camera; TC-cSO, True-color confocal scanning ophthalmoscope.

^*^ Grade of obscuration was defined as minimal for Grade 1 and significant for Grades 2–4.
